# Identification of factors for improved ethylene production via the ethylene forming enzyme in chemostat cultures of *Saccharomyces cerevisiae*

**DOI:** 10.1186/1475-2859-12-89

**Published:** 2013-10-01

**Authors:** Nina Johansson, Paul Quehl, Joakim Norbeck, Christer Larsson

**Affiliations:** 1 Department of Chemical and Biological Engineering, Chalmers University of Technology, SE412 96, Gothenburg, Sweden

**Keywords:** Yeast, *Saccharomyces cerevisiae*, Ethylene, Chemostat, Productivity

## Abstract

**Background:**

Biotechnological production of the traditional petrochemical ethylene is presently being explored using yeasts as well as bacteria. In this study we quantify the specific ethylene production levels at different conditions in continuous (chemostat) cultivation of *Saccharomyces cerevisae* expressing the ethylene forming enzyme (EFE) from *Pseudomonas syringae*.

**Results:**

Our study shows that oxygen availability is an important factor for the ethylene formation. Maintaining a high percentage dissolved oxygen in the cultivation was found to be necessary to achieve maximal ethylene productivity. Even at oxygen levels high enough to sustain respiratory metabolism the ethylene formation was restricted. Oxygen was also important for sustaining a high respiratory rate and to re-oxidize the surplus of NADH that accompanies ethylene formation. By employing three different nitrogen sources we further found that the nitrogen source available can both improve and impair the ethylene productivity. Contrary to findings in batch cultures, using glutamate did not give a significant increase in specific ethylene production levels compared to the reference condition with ammonia, whereas a combination of glutamate and arginine resulted in a strongly diminished specific ethylene production. Furthermore, from cultivations at different dilution rates the ethylene formation was found to be coupled to growth rate.

**Conclusion:**

To optimize the ethylene productivity in *S. cerevisiae* expressing a bacterial ethylene forming enzyme, controlling the oxygen availability and growth rate as well as employing an ideal nitrogen source is of importance. The effects of these factors as studied here provide a basis for an optimized process for ethylene production in *S. cerevisiae*.

## Background

Despite a general consensus that the dependency on finite resources such as oil and natural gases should be reduced, the consumption rate of these continues to increase [1]. The main usage of these resources is as fuels. However, a wide range of other chemicals are also produced from petroleum, among which the most abundant are hydrocarbon monomers such as ethylene (ethene, C_2_H_4_). Due to an increased usage in foremost Asia, the demand on ethylene has increased with an average of 2.5% per year in the last decades and in 2012 the global demand for ethylene was roughly 124 million tons per year [2-4]. The reason for this demand is the versatility of ethylene as a building block for a wide range of compounds, primarily its polymer product polyethylene which forms the basis for many plastic materials [5].

Biological production of ethylene could be an alternative to the traditional petroleum based chemical method. Several types of microorganisms, including both bacteria and fungi, have been reported to naturally produce ethylene [6]. Two different pathways for ethylene production have been identified within these organisms. One group produces ethylene via the KMBA pathway in which methionine is converted into ethylene in a two-step process, whereas the other group employs a single enzyme, the ethylene forming enzyme (EFE), to convert 2-oxoglutarate directly into ethylene [6,7]. Measuring the ethylene productivity of 757 bacterial strains, *Pseudomonas syringae pv. Phaseolicola* PK2 was found to be the most productive species. This strain was classified as belonging to the 2-oxoglutarate dependent group [7].

The major advantage of utilizing microorganisms for the production of chemicals is the possibility to exploit biomass, such as forest and agricultural residues, as raw material in the fermentative process. Biomass is a plentiful and renewable material and microorganisms can, via metabolic engineering, be altered to acquire the ability to transform the carbons (mainly sugars) in the biomass into desired products as reviewed by e.g. Nielsen et al. [8] and Zhang et al. [9]. On this basis, several biological systems for the conversion of sugars into ethylene have been examined. Heterologous ethylene production via mainly the *P. syringae* EFE has been reported for a variety of fungi [10,11] and bacteria [12-14]. A further alternative for biological ethylene production that has been investigated is the conversion of sunlight and CO_2_ into ethylene via cyanobacteria [15,16].

Some initial cultivation studies of these ethylene producers have been performed, primarily in shake flask and batch setup in bioreactors. Several of these studies have identified factors which could be restricting the ethylene reaction and hence negatively affect the ethylene yields. The most commonly mentioned factors are; oxygen availability [12,17], respiration rate [18] and substrate provision [10,12,16]. Since optimal production levels are required to make biological ethylene production a feasible alternative to the traditional petroleum based production method, each of these limitations must be studied to understand the extent to which they affect ethylene yield and thereby identify approaches for improving the ethylene production ability.

Using EFE expressed in the yeast *S. cerevisiae*, this study addresses the most commonly indicated limiting factors of biological ethylene production. A strain of the yeast *S. cerevisiae* engineered to express the *P. syringae* EFE was employed. *S. cerevisiae* was chosen due to the ease with which genetic manipulation can be performed in it, but also due to its relative sturdiness and the fact that it is already used as a biological factory in industrial settings [19]. Cultivations were performed in a chemostat mode to facilitate the comparison of different conditions. Oxygen level, respiration rate and substrate provision were all addressed in different experiments. Furthermore, strategies to increase respiration rate were evaluated for their effect on ethylene production levels.

## Results and discussion

Expression of the EFE-gene in *S. cerevisiae* has been shown to confer the ability to produce ethylene to the yeast [10]. Biochemical studies of the EFE have proposed that the enzyme performs the following reaction [20]:

(1) $$\begin{array}{l} 3\;2-\text{oxoglutarate}+{3\;O}_{2}+1\;\text{Arginine}\to 2\;\text{Ethylene}\\ \;+1\;\text{Succinate}+1\;\text{Guanidine}\\ \;+1\;\left(S\right)-1-\text{pyrroline}-5-\text{carboxylate}\;\left(P5C\right)\\ \;+{7\;\mathrm{CO}}_{2}+{3\;H}_{2}O. \end{array}$$

Theoretical modeling of this reaction together with the central carbon metabolism has proposed routes for improved ethylene production [18]. In this study we take into consideration a number of the factors suggested to affect the ethylene productivity. Each of the proposed substrates of EFE – 2-oxoglutarate, arginine and oxygen - have been studied with respect to their effects on ethylene formation using well defined chemostat conditions. We further investigated the effect of dilution rate and dissolved oxygen levels as well as the respiration rate.

### The effect of nitrogen source on ethylene production

It was found that changing the nitrogen source from ammonium to glutamate led to an increase in ethylene productivity with almost 50% compared to the standard condition with ammonium as nitrogen source (from 178 to 242 μg L_Culture_^-1^ h^-1^, see Table 1). However, the main origin of the increase in ethylene production was found to be an increase in biomass and the specific production rate only showed a very modest and non-significant increase from 30.4 to 32.3 μg g_DW_^-1^ h^-1^. This result is in contrast with the findings of Pirkov et al. [10] who showed that changing the nitrogen source from ammonium to glutamate significantly increased the specific ethylene productivity in batch cultivations.

**Table 1:** The effect of nitrogen source on the ethylene production

		**Ethylene**		
**N-Source**	**Biomass [g L**^**-1**^**]**	**Productivity [μg L**_**Culture**_^**-1**^**h**^**-1**^**]**	**Specific productivity[μg g**_**DW**_^**-1**^**h**^**-1**^**]**	**Yield [μg g**_**Glucose**_^**-1**^**]**
(NH_4_)_2_SO_4_ (7.5 g/L)	5.72 ± 0.95	178 ± 25	30.4 ± 2.8	164 ± 21
Glutamate (7.5 g/L)	7.48 ± 0.14	242 ± 2	32.3 ± 0.3	233 ± 0.8
Glutamate + Arginine (3.5 g/L each)	7.34 ± 0.03	101 ± 1	13.8 ± 0.2	96.8 ± 1.0

Ethylene production levels with different nitrogen sources. Values given are averages of two separate samples. ± indicate minimal/maximal levels of the two samples.

Chemostat cultivations at D=0.1 h^-1^ and with a glucose concentration of 10 g L^-1^.

Studies on chemostat cultivations of *S. cerevisiae* performed by Boer et al. [21] suggested that, in glucose limited continuous cultures, arginine might be a limiting metabolite. As arginine is essential for the EFE reaction [20] experiments were performed in which the nitrogen source was a combination of 50 w-% (3.5 g L^-1^) arginine and 50 w-% (3.5 g L^-1^) glutamate. This combination of glutamate and arginine as nitrogen sources led to a significant increase in biomass formation, similar in size to that on glutamate (Table 1). As the carbon skeleton of both glutamate and arginine can be used as carbon and energy source, this increase in biomass was expected. However, an unexpected finding was that the combination of arginine and glutamate led to a significant decrease in specific ethylene productivity, which dropped to 13.8 μg g_DW_^-1^ h^-1^. This corresponds to approximately 45% of the specific ethylene production levels observed with ammonia as a nitrogen source. One possible explanation for the decreased ethylene production could be coupled to the function of the EFE. Commonly the reaction of the EFE is given in a summarized form as shown above (reaction 1). However, Fukuda et al. [20] proposed that the EFE functions in a “dual circuit” fashion in which reaction (1) is actually a mean value of two different reactions, an ethylene forming (EC 1.13.12.19, see http://www.brenda-enzymes.org/php/result_flat.php4?ecno=1.13.12.19) and a non-ethylene forming (EC 1.14.11.34, see http://www.brenda-enzymes.info/php/result_flat.php4?ecno=1.14.11.34):

(2) $$\begin{array}{l} 2‒\mathrm{oxoglutarate}+O_{2}\to \text{Ethylene}\\ \;+{3\;\mathrm{CO}}_{2}+H_{2}O \end{array}$$

(3) $$\begin{array}{l} 2‒\mathrm{oxoglutarate}+\mathrm{Arginine}+O_{2}\to \mathrm{Succinate}\\ \;+\mathrm{Guanidine}+P5C+{\mathrm{CO}}_{2}+H_{2}O \end{array}$$

The two reactions have been reported to occur at an average ratio of 2:1, using purified enzyme. It is possible that by supplying extra arginine we could be pushing the balance between the two reactions towards the non-ethylene forming reaction (reaction 3), in which arginine is a substrate instead of a co-factor. Hence, less ethylene would be formed per glucose which would in turn explain the decreased ethylene formation found in this study when combining arginine and glutamate as nitrogen source.

### Effect of oxygen availability on ethylene production

Fukuda et al. [22] identified oxygen as an essential factor for the ethylene formation *in vitro* using purified EFE. Several later studies have also proposed oxygen provision as a limiting factor for ethylene formation via the EFE *in vivo*[10,12,18]. To elucidate the effect of oxygen availability on the ethylene formation via the EFE we also investigated the effect of dissolved oxygen concentration on the ethylene productivity.

It was found that an alteration in dissolved oxygen levels was followed by a relatively fast response (within the interval of sampling, which was one hour for this experiment), in which an increase in oxygen level was followed by an elevation of specific ethylene production, and a decrease in oxygen level was followed by a reduction in ethylene production.

**Figure 1:**
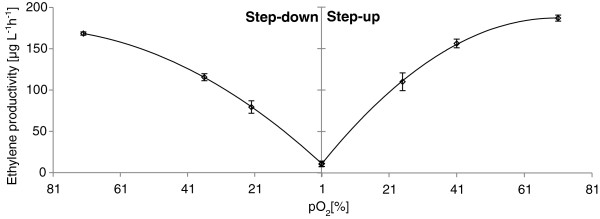
**The effect of dissolved oxygen on ethylene productivity in *S. cerevisiae *expressing the pYX212-EFE construct.** Continuous cultivation performed at dilution rate 0.1 h^-1^ in defined media with 10 g glucose L^-1^. The dissolved oxygen was step-wise decreased then stepwise increased. 100% pO_2_ is equal to the amount dissolved oxygen in air saturated water at 30˚C.

To more fully understand the correlation between ethylene production and oxygen availability, several different levels of oxygenation were tested. Alterations of percentage dissolved oxygen were performed stepwise from a high level (pO2 > 70%) via two intermediate levels to a very low level (pO_2_ = 1%), then increased back up to a high level via two intermediate levels. In each case steady-state was established before changing to the next level. The effect on ethylene formation was notable, in that the steady-state levels of ethylene production showed a clear positive correlation to the dissolved oxygen level (Figure 1). This pattern agrees with previous findings of effects of dissolved oxygen on ethylene formation in *P. syringae* cultures [23]. The ethylene production during the step-up experiments was somewhat higher than at corresponding pO_2_ level during the step-down experiment, indicating that some adaptation might have occurred. The step–up experiment further revealed an upper threshold for the oxygen above which there seems to be little further effect on ethylene formation (Figure 1). This level was found to be around a pO_2_ value of approximately 70%. In later experiments it was further confirmed that an oxygenation level around 90% did not give any added increase in ethylene production (data not shown). Research on enzymes of the same family as EFE, the Fe(II)/α-ketoglutarate dependent hydroxylases or 2-oxoglutarate-Fe(II) oxygenase family, has shown that if the central Fe(II) ion, which coordinates the binding of the substrates to the enzyme, becomes oxidized to Fe(III) the enzyme is inactivated [24]. A too high oxygen pressure can hence have a limiting effect on the ethylene formation. However, in contrast with the findings of Hahm et al. [23] we did not see that an oxygenation above 90% led to a decrease in ethylene production compared to the levels attained at a pO2 level just above 70% (data not shown).

Previous studies of continuous cultures of *S. cerevsiae* have indicated that even very low percentages of oxygen can sustain full respiration at a dilution rate of 0.1 h^-1^[25,26]. Accordingly, the biomass formation was found to be constant over the full range of dissolved oxygen percentages applied. Furthermore, no significant production of ethanol or glycerol could be detected. Therefore, the effect on ethylene formation found in this study can be coupled directly to the oxygen availability for the enzyme rather than a major metabolic shift of the yeast. This indicates that the affinity of EFE for oxygen is low and a good oxygen provision must therefore be ensured for optimal ethylene production. This has implications for how cultivations should be set up, especially when performing studies of ethylene production in yeast strains using shake flasks, where oxygen levels will decrease as cell densities increase. In this case, care should therefore be taken to either ensure proper aeration or to work at low cell densities.

### The effect of growth rate and respiration rate on ethylene production

**Figure 2:**
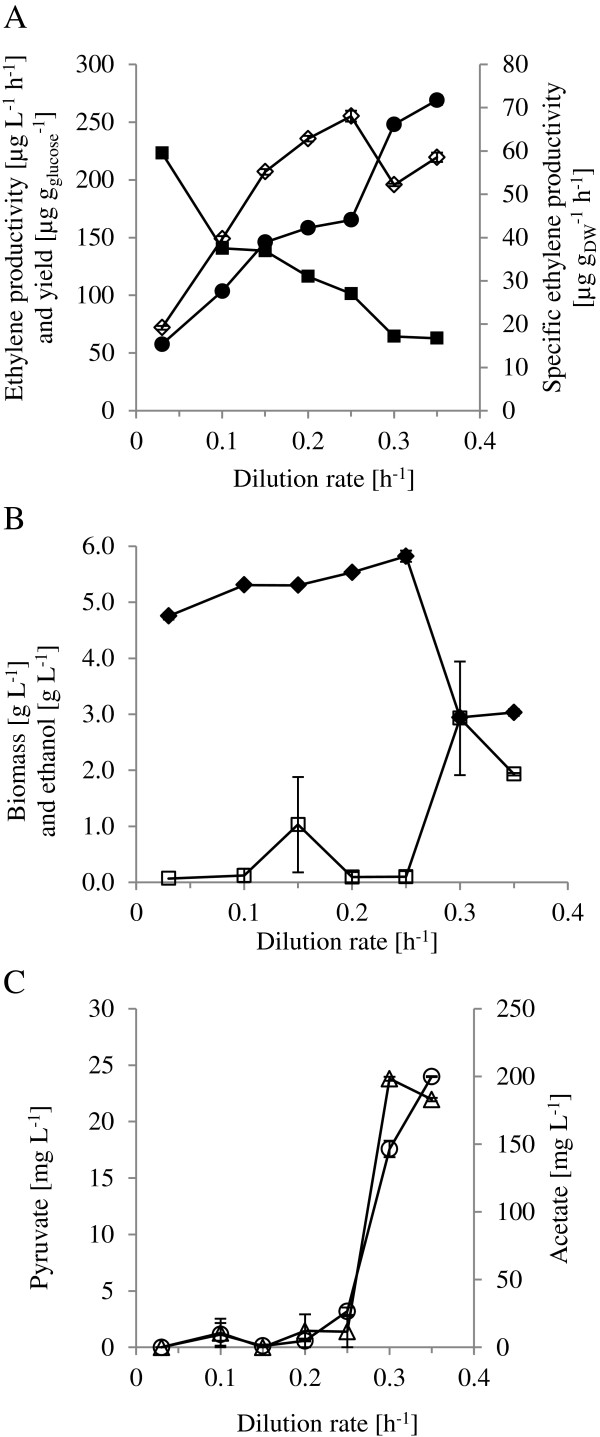
**Growth and product characteristics at different dilution rates for the *S. cerevisiae *strain expressing the EFE.** Cultures were performed in defined minimal media with 10 g L^-1^ glucose. Dilution rates were varied from 0.033 h^-1^ to 0.35 h^-1^. **A)** Ethylene productivity (open diamond), specific productivity (closed circle) and yield per gram glucose (closed square). **B)** Biomass (closed diamond) and ethanol (open square). **C)** Pyruvate (open circle) and acetate (open triangle).

Growth rate, which can easily be set in a chemostat culture by varying the dilution rate, has a strong influence on gene expression and the metabolism (e.g. the degree of respiration) [27,28]. To evaluate the effect of metabolism and growth rate on ethylene production, we performed continuous cultures at different dilution rates, ranging from very low levels where the metabolism is purely respirative (0.033 h^-1^), to relative high levels (0.35 h^-1^) where the yeast grows respiro-fermentatively. A dissolved oxygen percentage of approximately 80% was maintained throughout the entire dilution rate series to ensure that oxygen would not be a limiting factor. Growth characteristics and metabolite levels at the different dilution rates are presented in Figure 2.

In general, the specific ethylene production rate (μg_ethylene_ g_DW_^-1^ h^-1^) increased with increased dilution rate, as can be seen in Figure 2A. Between the dilution rates of 0.25 h^-1^ and 0.3 h^-1^ a noticeable jump in specific productivity can be seen. At this point the yeast switches to respiro-fermentative growth. Indicative of this switch is the decrease of biomass formation and the onset of ethanol formation, both of which can be seen for the cultivations (Figure 2B). A further indicator of the shift to respiro-fermentative growth is the overflow of pyruvate from the glycolysis which was also noticed in these cultures. As the dilution rate was increased to 0.30 h^-1^ there was, moreover, a sharp increase in extracellular acetate concentration, also typical for the shift to partly fermentative growth in *S. cerevisiae*. This point of respiro-fermentative onset correlates with previous found values for *S. cerevisiae*[29,30], indicating that the EFE does not impose any drastic metabolic burden on the cell.

Furthermore, each increase in dilution rate was linked to an increase in specific glucose uptake rate (data not shown). As the glucose uptake rate increased more than the ethylene productivity for each increase in dilution rate, the ethylene yield (μg_ethylene_ g_glucose_^-1^) declined over the dilution rate span employed (Figure 2A).

The flux balance analysis of ethylene production in *S. cerevisiae* performed by Larsson et al. [18] indicated that respiration and the consumption of NADH are limiting factors for the formation of ethylene. The reason being that several of the reactions close to the EFE are NADH coupled. Hence, an increased respiration rate could be advantageous for the ethylene formation. It was previously shown that addition of benzoate to the media significantly increased the specific oxygen uptake rate and specific glucose flux [31,32]. In the study by Verduyn et al. [31] the addition of benzoate increased the respiratory capacity of *S. cerevisiae* almost eight times up to 19.5 mmol O_2_ g_DW_^-1^ h^-1^ at a dilution rate of 0.1 h^-1^. The effect of benzoic acid is linked to it functioning as an uncoupler by affecting the cytosolic proton concentration. This will lead to an increased energy demand which can be observed via a reduction in biomass [31]. In our cultures a drastic decrease in biomass, from 5.7 g L^-1^ to 0.72 g L^-1^, was seen with 7.5 mM benzoate. Similar to the experiment by Verduyn et al. [31] an increase in specific pyruvate formation, compared to the culture without added benzoate, was seen and a significant residual glucose concentration could be detected. The specific production rate (μg g_DW_^-1^ h^-1^, Table 2) of ethylene with benzoic acid was significantly higher than in the reference cultures, indicating that an increased respiration rate indeed is positive for the ethylene formation. However, due to the decreased biomass the productivity (μg L^-1^ h^-1^, Table 2) was low.

**Table 2:** The effect of respiration rate on ethylene formation

**Condition**	**Ethylene**	
	**Specific productivity [μg g**_**DW**_^**-1**^**h**^**-1**^**]**	**Productivity [μg L**_**Culture**_^**-1**^**h**^**-1**^**]**
Reference condition	30.4 ± 2.8	178 ± 25
+ 7.5 mM Benzoate	50.3 ± 1.3	37.7 ± 0.9
+ 1 mM Azide	0	0

Ethylene production levels in cultures with varied respiration rate.

Values given are averages of two separate samples. ± indicate minimal/maximal ethylene levels of the two samples. Reference condition is performed in defined media with 10 g glucose and 7.5 g (NH_4_)_2_SO_4_ per liter. Benzoate increases respiration, whereas azide inhibits the respiration. These were added to the same media used in the reference condition. All cultivations were performed at D=0.1 h^-1^ and pH 5.0.

To further investigate the correlation between respiration and ethylene formation, we performed cultivations with sodium azide, which effectively blocks the respiration [33]. When azide was added to the culture ethylene formation was immediately prohibited, which is a further indication of the close correlation between respiratory rate and ethylene formation in *S. cerevsiae*.

The results presented above suggested that increasing the respiratory rate of *S. cerevisiae* should result in higher ethylene yields, however the increased respiration rate should not come at too high an expense for the cell. One alternative could be to use the TM6* strain of *S. cerevisiae* which contains a chimeric point mutated version of the Hxt1 and Hxt7 glucose transporters. This strain seems to lack the Crabtree effect and respires even at high external glucose levels. It also respires at a rate 4.5 times higher than the reference stain [34]. Another alternative could be to alter the redox balance by using only the NAD-dependent glutamate dehydrogenase (GDH2), as suggested by Larsson et al. [18].

All of these strategies could increase the ethylene yield significantly, however the theoretical yield is still quite low. Larsson et al. [18] reported it to be 2 mol ethylene / 100 mol glucose in their study. The economic feasibility of this system is hence questionable, at least as long as the oil price remains low.

## Conclusions

In summary, this study investigated the effects of several different cultivation factors on ethylene formation in *S. cerevisiae* expressing the EFE in continuous cultures. Our main finding is that oxygen availability is crucial for ethylene production. This can be coupled to two reasons: firstly, most likely EFE has a low affinity for oxygen and secondly, the requirement of oxygen for oxidation of NADH in respiration. Linked to the functioning of the EFE we also suggest that elevated arginine levels will shift the reaction towards the unwanted side reaction of EFE (reaction 3), which suggests that a process and strain optimized for ethylene production via the EFE should strive to keep intracellular arginine levels low. The ability to consume NADH seems to be a crucial requirement for optimal ethylene production, both from the previous metabolic modeling and from the data presented in this paper. An ethylene production fermentation process and strain should therefore address this issue, e.g. by ensuring a high degree of respiration and/or by changing the co-factor utilization of Gdh.

## Materials and methods

### Yeast strain construction

The plasmid construct (pYX212-EFE) in which the open reading frame of the EFE of *Pseudomonas syringae pv. phaseolicola* is cloned after the TPI1 promoter [10] was introduced into the *S. cerevisiae* strain CEN.PK 113-5D (*MATa MAL2-8c SUC2 ura3-52*).

### Cultivation conditions

Aerobic chemostat cultivations were performed in carbon limited CBS minimal media as reported by Verduyn et al. [31] with 3.5 g KH_2_PO_4_ and 0.75 g MgSO_4_*7H_2_O per liter and double amount of trace metals. As standard condition 7.5 g ammonium sulfate and 10 g glucose per liter was used. 100 ml overnight shake flask culture in the CBS media was used as inoculum for the bioreactor. The cultivations were performed in a 3 L Belach BR02 fermentor (Belach, Stockholm, Sweden) with a working volume of 2 L, operated at 30°C and with a standard aeration rate of 1.0 L min^-1^. The pH was kept constant at 5.0 using 1 M or 2 M NaOH.

In the initial phase the bioreactor culture was run as a batch cultivation. The switch to continuous mode was done as the batch cultivation reached late exponential phase.

Dilution rate was based on earlier measurements of pump performances. By weighing the amount of media which was pump at a certain rpm setting during a specific time period and then repeating this over a wide range of rpm settings an equation was found to calculate media flow through the pump based on the rpm. As the volume of the cultivation was known the dilution rate could be altered according to wishes. Routinely a dilution rate of 0.1 h^-1^ was used. A control experiment was performed to verify that the dilution rate did not affect the stripping of ethylene from the culture. The measured ethylene concentration in the off gas was identical at all conditions tested, i.e. from no dilution up to a dilution rate of 0.35 h^-1^.

The effect of oxygen availability on ethylene production was considered by altering the dissolved oxygen percentage. Desired oxygenation level was achieved by decreasing the stirring rate, reading the pO_2_ value and if necessary adjusting the rpm further. Stirrer speed was verified to not affect the stripping of ethylene from the culture over the range used. To examine the effect of nitrogen source on ethylene production, cultivations were performed with 7.5 g glutamate per liter or 3.5 g glutamate plus 3.5 g arginine per liter as alternatives to ammonium sulfate. Further, cultivations were performed at several dilution rates, from 0.033 h^-1^ to 0.35 h^-1^.

### Ethylene measurement by GC-FID

Ethylene production was measured on-line by connecting the off gas to a HP 5890II GC-FID (Hewlett-Packard, USA) equipped with a HayeSep Q 80/100 porous packed column. The GC was operated isothermally at 60°C with injection temperature 70°C and detection temperature 125°C. Helium was used as carrier gas at a flow rate of 30 ml min^-1^ and the flame was fed with hydrogen gas. Samples were compared to an ethylene standard to determine amounts. Samples were analyzed using ChromNav (JASCO, Japan). Samples were taken automatically every second hour as standard setting.

### Analysis of biomass

Cell growth (biomass) was followed by measuring OD at 610 nm. Dry weight was determined at two separate time points for each condition by the method described by Dynesen et al. [35]. Nitrocellulose filters with pore size 0.2 μm (Sartosius Stedim Biotech, Göttingen, Germany) were pre-dried 20 min at 119 W and then weighed. A known sample volume was filtered through the pre-weighed filter papers using water suction and then washed twice with deionized water. Filters with culture sample were microwaved 20 min at 119 W, stored in a desiccator for a minimum of 24 hours before being weighed and dry weight determined.

### Analysis of extracellular metabolites

Extracellular samples were collected by extracting samples from the cultivation and directly centrifuging them at 13 000 x g for 2 min. Supernatants were stored at -20°C until measured. Glucose, ethanol, glycerol, acetate, pyruvate, succinate and 2-oxoglutarate were measured using HPLC. Two separate samples were taken, with a time interval, for each condition. Ethanol was not corrected for evaporation.

## Competing interests

The authors declare that they have no competing interests.

## Authors’ contributions

NJ and PQ performed all the experiments and took part in designing the study, evaluating the results and writing the manuscript. JN and CL took part in designing the study, evaluating the results and writing the manuscript. This manuscript has been approved by all the authors listed.
